# Why unequal AI access enhances team productivity: the mediating role of interaction processes and cognitive diversity

**DOI:** 10.3389/fpsyg.2025.1636906

**Published:** 2025-09-08

**Authors:** Jiaxuan Han, Ruqin Ren

**Affiliations:** USC-SJTU Institute of Cultural and Creative Industry, Shanghai Jiao Tong University, Shanghai, China

**Keywords:** AI access, cognitive diversity, team interaction processes, team productivity, human-agent teams, human-AI collaboration

## Abstract

**Introduction:**

Generative artificial intelligence (GenAI) is widely viewed as valuable for improving the performance of human-agent teams (HATs). However, in reality, not all members have equal access to AI tools, making uneven AI integration an important factor impacting team composition and, thus, team effectiveness. While unequal access might seem detrimental, potentially hindering technology utilization, it could also foster deeper interactions and diverse expertise. To clarify these mechanisms, this study extends the classic Input-Mediator-Output model to an Input-Process-State-Output (IPSO) framework.

**Methods:**

A lab experiment involving 60 two-person teams was conducted, with teams assigned to unequal, full, or no AI access conditions.

**Results:**

The findings indicate that unequal AI access yields the highest productivity, improving both task quality and completion time compared to no or full AI access. This effect is driven by two key mechanisms. First, negative socio-emotional interactions and increased cognitive diversity serve as a positive serial mediation pathway linking unequal AI access to enhanced task quality. Second, unequal AI access leads to more concentrated and imbalanced questioning behaviors, which accelerates task completion.

**Discussion:**

This study provides an in-depth theoretical explanation of how AI integration structures operate in HATs and offers a foundation for strategically optimizing GenAI access in human-agent teaming.

## 1 Introduction

As generative AI (GenAI) technology continues to evolve, more individuals and organizations are integrating GenAI into collaborative work, forming Human-Agent Teams (HATs). HAT refers to a collaborative effort between one or more humans and autonomous agents to achieve a common goal (McNeese et al., 2018; O’Neill et al., 2022). A recent industry report found that 78% surveyed organizations adopted AI in their organizations, with 56% of employees directly engaging with AI tools to automate or augment job tasks (BusinessWire, 2023). Despite GenAI’s widespread application, challenges remain—particularly regarding the often complex and inconsistent ways team members adopt AI. It cannot be taken for granted that AI access among team members is equal. In practice, some team members use GenAI extensively, while others lack access or proficiency (Humlum and Vestergaard, 2025), resulting in diverse AI integration structures within Human-Agent Teams.

The challenge of inconsistent AI access is particularly salient in short-term project-based team settings, which are often termed *ad hoc* or temporary teams (Finholt et al., 2014; Liu et al., 2004; Majchrzak et al., 2012). Unlike long-standing corporate teams, people in temporary teams lack prior relationships and must collaborate effectively with minimal knowledge of each other. In these settings, AI tools become important external resources. Moreover, as temporary teams typically lack clearly specified management hierarchies or power structures (Stone et al., 2010), technological asymmetries may carry greater weight in shaping team dynamics. Thus, the uneven distribution of AI access raises important questions about how different GenAI access patterns affect already complex and challenging temporary team collaboration.

Extant literature has demonstrated that AI adoption influences team productivity, which is defined as the collective effectiveness (i.e., task quality) and efficiency (i.e., task time) (Hackman, 1978; Kozlowski and Ilgen, 2006; Kwarteng et al., 2023; Noy and Zhang, 2023). However, how and why GenAI integration structures might influence team productivity remains a subject of theoretical debate. Though it seems intuitive to assume that equipping all members with the most advanced technology would be optimal, given widely existing evidence that GenAI usage increases individual users’ creativity and productivity (Cui et al., 2024; Doshi and Hauser, 2024; Noy and Zhang, 2023). Limiting AI access may also result in imbalanced participation and decreased morale and contribution from those without access (Bayerl et al., 2016; Rogers et al., 2009; Simaremare et al., 2024). However, there also exist counterarguments that limiting AI touchpoints may enhance team interactions (Li et al., 2024; Raisch and Krakowski, 2021) and encourage diverse perspectives to emerge as the team could tap into both personal expertise and AI outputs, rather than having all members quickly converging on the same AI-generated outputs (Doshi and Hauser, 2024).

To resolve conflicting views on the optimal strategy for GenAI adoption in HATs, the current study explores how full AI access, partial AI access, and no AI access shapes team dynamics differently, and how these dynamics, in turn, influence collaborative performance. Unequal AI access is of particular interest as it introduces distinct intra-team dynamics that are less likely to emerge in uniformly equipped teams, including asymmetric information distribution (Sebo et al., 2018; Gurkan and Yan, 2023; Zvelebilova et al., 2024), divergent expectations of contribution (Doshi and Hauser, 2024; Stasser and Titus, 2003; Lu et al., 2012), and shifts in perceived social status (Rogers et al., 2009; Meeussen and Van Dijk, 2016). Such dynamics represent novel organizational conditions that may fundamentally reshape how teams interact, adapt, and perform. Despite its increasing relevance, prior research has primarily contrasted teams with full AI access and those without (e.g., Han et al., 2024; Gurkan and Yan, 2023), overlooking this nuanced middle ground. The findings illuminate both the practical implications of AI integration in teamwork and the theoretical significance of how unequal access reshapes team interaction and productivity.

We draw on O'Neill et al. (2023)‘s recent extension of the classic Input–Mediator–Output (IMO) model (Hackman, 1978; Ilgen et al., 2005; Marks et al., 2001). The IMO model has historically been used in research on human team effectiveness and small group interactions (Hackman, 1978; Steiner, 1972; Ilgen et al., 2005; Marks et al., 2001), providing a structured lens to examine how team inputs (e.g., member composition, task design) influence team outputs (e.g., performance, satisfaction) via mediating mechanisms such as team processes and emergent states. O'Neill et al. (2023) applied the IMO model HATs, providing a framework for examining how inputs unique to HATs—such as different modes of human-AI composition—shape mediating team dynamics and ultimately affect outcomes. To better adapt this umbrella framework to our research context, we now propose two conceptual modifications to explain how AI integration patterns (input) affect team productivity (output) in greater detail.

First, regarding team *input*, we conceptualized varied AI integration patterns as a key team composition factor. Team composition is the different ways that human-autonomy is combined in HATs. Most existing studies treat AI usage as a binary input—either present or absent (Al Naqbi et al., 2024; Gohar and Utley, 2023; Gurkan and Yan, 2023; Han et al., 2024), overlooking the nuanced AI integration structures that more accurately reflect real-world practices. For example, Han et al. (2024), in their examination of the effects of GenAI on team collaboration in creative tasks, included only two conditions: human teams with GenAI and without GenAI. Similarly, Gurkan and Yan (2023) designed their experiment such that a chatbot provides information in a group chat without engaging in direct interaction, considering only the presence or absence of AI when evaluating its effects on cognitive diversity and team decision-making. Such designs oversimplified the patterns of GenAI allocation among team members. To better capture the nuances of AI adoption in reality, we aim to explore how AI integration structures as a team input impact team processes and outcomes by carefully considering three conditions of AI integration: no access to AI, partial access to AI, and full access to AI among the team.

**Figure 1:**
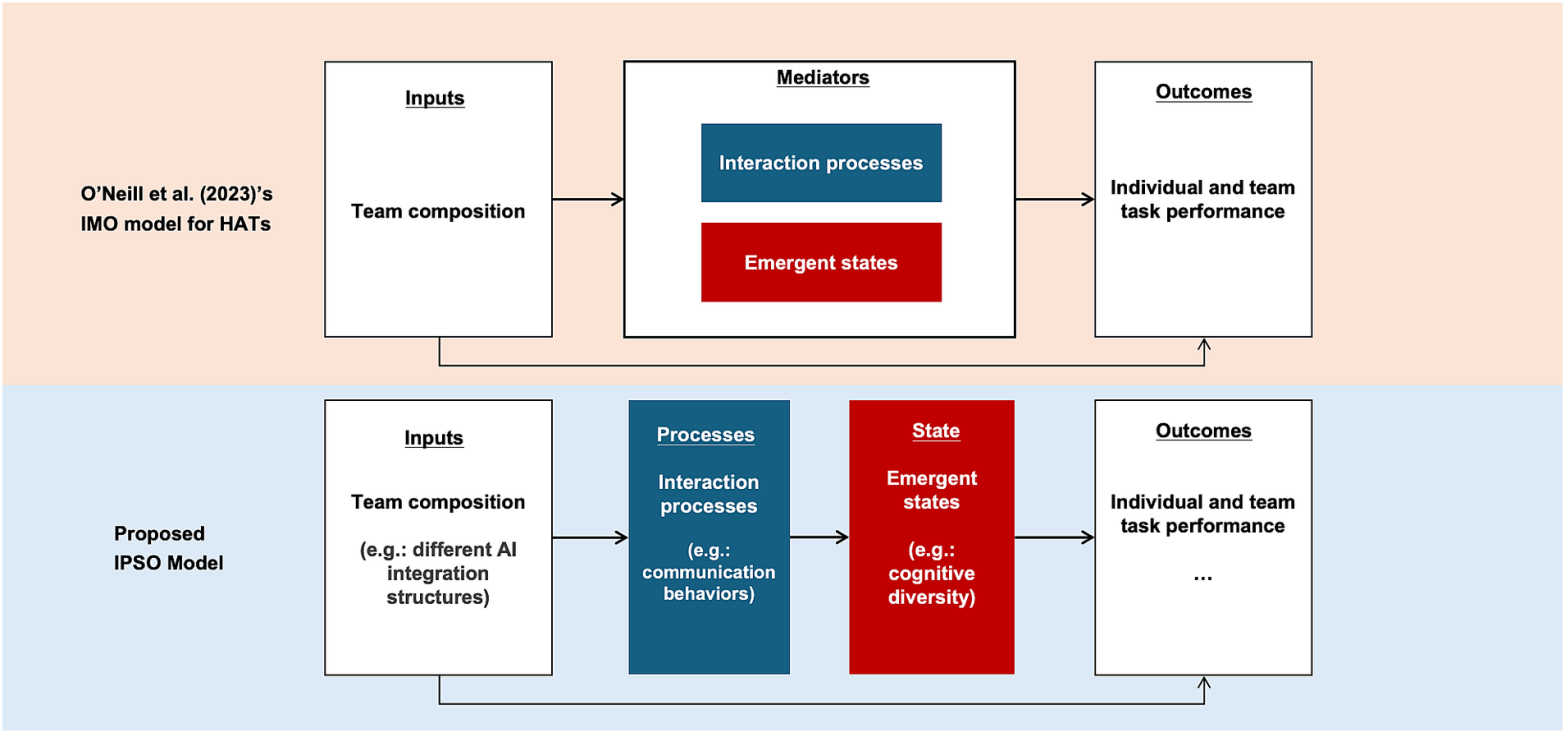
IPSO model: an extended framework for O'Neill et al. (2023)’s IMO model for HATs.

Second, for the *mediator* part, O'Neill et al. (2023) emphasized the importance of considering mediating mechanisms and moving beyond a simplistic independent-dependent variable modeling approach. In their framework, the mediator was conceptually divided into two broad categories: interaction processes (e.g., planning, communication, coordination) and emergent states (e.g., trust, shared mental models, situation awareness, or affective states). However, they did not specify the potential relationships between these two types of mediators. We further propose a sequential relationship between them: interactions processes, as manifested by individual members’ communication behaviors, give rise to emergent states (cognitive or affective) at the team level. In other words, emergent states are not static but dynamically shaped through interactions. Thus, we delineate the mediator part into two consecutive steps and propose them as chained mediators, transforming the *Input-Mediator-Output (IMO)* model into an *Input-Process-States-Output (IPSO)* model, which we then subject to empirical testing. Figure 1 illustrates how we further modify the IMO model for HATs proposed by O'Neill et al. (2023).

For this current study, we focus on communication behaviors as the ‘Process’ factor and cognitive diversity as the ‘State’ factor in our IPSO model. While cognitive diversity is a classic construct in teaming research and is often recognized as a team emergent property evolving through dynamic interactions (Marks et al., 2001; Mello and Rentsch, 2015), and some initial HATs research links AI usage to cognitive diversity (Gurkan and Yan, 2023), these studies often stop short of identifying specific interaction behaviors that mediate this relationship. We will use Bales’ Interaction Process Analysis (IPA) to classify four specific types of communication behaviors and explore how they potentially alter task-related information flow and contribute to cognitive divergence among members.

In conclusion, our IPSO model aims to provide a more accurate description of how different GenAI access structures influence team interaction patterns and cognitive diversity, and how these factors jointly impact team outcomes such as task quality and completion time. Accordingly, we attempt to address this general question:

*How do* var*ied GenAI integration structures affect team productivity via the serial mediation mechanisms of team interaction behaviors and cognitive diversity?*

## 2 Hypotheses development

### 2.1 The paradox of unequal AI integration

Recent studies have consistently shown that integrating AI into human teams can enhance collaborative outcomes by fostering creativity (Jeong and Jeong, 2024), improving decision-making (Gurkan and Yan, 2023), and boosting productivity (Al Naqbi et al., 2024). However, moving beyond this binary perspective of AI adoption, real-world scenarios often involve uneven access to AI within teams. When examining how such unequal distribution of this emerging technology affects team productivity, prior research offers conflicting conclusions. The *positive perspectives* suggest that limiting AI access to some of the team members facilitates more focused and interactive use of GenAI, thus enhancing its utilization depth and maximizing its potential (Raisch and Krakowski, 2021). This close human-AI collaboration can foster creativity and improve task quality (Zhang et al., 2025). Additionally, the selective use of GenAI by only some team members helps to generate diverse cognitive inputs and reduce homogeneous ideas (Doshi and Hauser, 2024), teams thus may achieve high levels of creativity by building a wider pool of expertise that is differentiated and specialized (Zhang et al., 2025). With respect to task time, unequal AI access can shorten completion time by streamlining communication and facilitating strategic adjustments (Li et al., 2022), as full access may increase coordination complexity with many more human-AI pairings to manage (Becker et al., 2008). Limiting such human-AI combinations can reduce communication costs and accelerate task execution. Moreover, the diverse inputs resulting from unequal access to technology can make teams more flexible and agile (Pieterse et al., 2011), allowing them to adjust more quickly in the face of change and unexpected situations (Harrison et al., 2000).

The *opposing viewpoint* suggests that full access to new technology is more beneficial for task quality because it fosters equal participation among team members (Rogers et al., 2009), potentially maximizing each individual’s contribution to the team (Li et al., 2024). When technology distribution is not equal, those without AI access may feel marginalized, which can diminish their motivation to participate and contribute actively (Bayerl et al., 2016), ultimately leading to lower overall team cohesion and reduced task quality. Furthermore, uneven AI distribution may prolong task completion time by increasing the difficulty of managing conflict and interpersonal tension caused by unequal participation among team members (Bankins and Formosa, 2023; Rogers et al., 2009). In addition, the diverse perspectives generated by varying collaboration patterns often require more extensive integration efforts to reach a consensus (Sauer et al., 2006), all of which demand additional time (Mohammed and Schillinger, 2022; Narayan et al., 2021). Therefore, we propose a set of competing hypotheses:

*H1a*: Teams with *unequal* AI access have greater team productivity (i.e., better task quality and faster task completion time) compared to those with no access or full access.

*H1b*: Teams with *full* AI access have better team productivity (i.e., better task quality and faster task completion time) compared to those with no access or unequal access.

### 2.2 The mediator role of cognitive diversity between AI integration and team productivity

Building on the above discussion, a likely mechanism through which AI integration structures influence team productivity is the diverse task-related perspectives and contributions that stem from differences in access, a concept commonly referred to as cognitive diversity. It is defined as the range of information, information processing styles, and perspectives of members, which is dynamically and interactively generated through communication (Gurkan and Yan, 2023; Sauer et al., 2006). Though cognitive diversity is a complex construct and has been defined in many varied ways (Kurtzberg, 2005; Sauer et al., 2006; Shin et al., 2012; Miller et al., 1998; Mohammed and Ringseis, 2001), Mello and Rentsch (2015) proposed a stability-based framework that categorizes cognitive diversity into four types, ranging from the most stable to the most malleable: trait-like (stable and consistent personal characteristics), developmental (which evolve over time but change gradually), acquired (context-dependent and flexible, such as task-related knowledge or attitudes), and exposed (the most malleable, shaped by specific experimental conditions). Our study specifically focuses on *acquired* cognitive diversity, which evolve dynamically with team context. This form of cognitive diversity is important for understanding team collaboration in our research context, given its direct susceptibility to variation in members’ access to external information sources, particularly AI technology and how it is integrated within teams.

We speculate that not distributing AI access equally within teams could lead to increased cognitive diversity mainly by triggering task-related information asymmetry and social status and role differentiation. First, unequal AI integration reshapes how information is accessed and shared within teams, leading to differences in members’ task-related information processing and perspectives (DeSanctis and Poole, 1994; Zhang et al., 2025). When only some team members have access to AI assistance while others do not, they are exposed to different sources of task-relevant information. AI-equipped members may form task opinions based on algorithmic interpretations or AI-generated contents (Gurkan and Yan, 2023; Sebo et al., 2018; Zvelebilova et al., 2024), whereas non-AI users rely on human discussions, intuition, or personal experience. In contrast, teams with full AI access could use highly similar information, as members largely depend on the homogenized outputs generated by AI (Doshi and Hauser, 2024). According to social confirmation bias, this shared information often overshadows unique insights derived from individual knowledge or experience (Lu et al., 2012; Stasser and Titus, 2003), easily results in convergent perspectives within the team. Therefore, unequal AI access is likely to foster greater cognitive diversity by generating a wider range of opinions arising from distinct informational environments.

Second, AI access serves as a substitute for human expertise (Doshi and Hauser, 2024; Korzynski et al., 2023; Noy and Zhang, 2023; Zhang et al., 2023), creating role and status differentiation between users and non-users. People with GenAI access may perceive themselves—and be perceived by others—as more competent due to their technological advantage (Meeussen and Van Dijk, 2016; Rogers et al., 2009). Drawing on status characteristics theory (Berger et al., 1980; Correll and Ridgeway, 2003), AI access could serve as a salient status characteristic, shaping interaction patterns and authority structures within teams (Zhang et al., 2025). High-status individuals typically make strategic decisions, while lower-status members focus on operational aspects of the task (Bunderson and Reagans, 2011). Such differentiated roles and statuses—emerging from unequal AI access—further contribute to more varied information processing styles and task-related perspectives among team members (Mello and Rentsch, 2015).

As the critical team-level psychological outcome of unequal AI access, increased cognitive diversity is commonly related to both positive and negative team-level outcomes (Horwitz and Horwitz, 2007; Simons and Rowland, 2011), such as task quality (Gomez and Lazer, 2019; Joniaková et al., 2021; Patrício and Franco, 2022; Schumpe et al., 2023) and task time (Harrison et al., 2000; Li et al., 2022; Mohammed and Schillinger, 2022; Sauer et al., 2006). We, therefore, posit it as a mediator between AI integration and team productivity without predicting directionality. It is hypothesized that:

*H2*: Cognitive diversity mediates the relationship between AI integration structure and team productivity.

### 2.3 The mediator role of team interaction processes between AI integration and cognitive diversity

Team interaction, as a dynamic process central to team functioning, plays a critical role in shaping emergent states such as cognitive diversity (Marks et al., 2001; Mello and Rentsch, 2015). Prior sections discussed how unequal AI access may create informational asymmetry and status differentiation within teams. These effects can directly alter how members exchange information and relate to one another (Ward, 2013), thereby affecting both cognitive diversity and productivity. To further unpack team interactions as observable actions, this study adopts Bales’ Interaction Process Analysis (IPA) framework (Bales, 1950; Nam et al., 2009; Soukup et al., 2020), a well-established categorizing scheme for team interactions. IPA separates complex team interactions into socio-emotional (positive/negative reactions) and task-related (questions/answers) domains, providing a structured approach for analyzing how different interaction patterns emerge under varying GenAI access conditions.

#### 2.3.1 Unequal AI access’s impact on socio-emotional area interactions

Socio-emotional interactions can be further divided into positive and negative reactions. Positive reactions include showing solidarity, releasing tension, and expressing agreement, while negative reactions refer to disagreement, tension, and antagonism (Nam et al., 2009). Unequal AI access can shape these emotional reactions in contrasting ways—potentially suppressing supportive behaviors due to perceived unfairness while at the same time encouraging disagreement as a result of divergent informational inputs (Mannes et al., 2014; Pelled et al., 1999).

First, perceived inequality in the distribution of a highly desirable technology may lead to misunderstanding and mistrust, thereby reducing the expression of positive interactions like support or agreement (Cronin et al., 2011; Kennedy and Pronin, 2008). This undermines the development of a psychologically safe environment that encourages broad participation and open perspective-sharing, ultimately hindering the emergence of cognitive diversity (Isohätälä et al., 2020). For example, repeatedly interrupting others’ views during group discussions may trigger defensiveness and discourage the contribution of diverse ideas. Thus,

*H3a*: Unequal AI access *reduces positive* socio-emotional behaviors, which in turn influence cognitive diversity.

Second, unequal AI integration can increase negative reactions such as disagreement by encouraging the exchange of unique and unshared information from distinct perspectives (Lu et al., 2012)—AI users draw on system-generated content, while non-users rely more on personal experience. When team members challenge one another’s assumptions or interpretations, they may surface divergent mental models and expose hidden knowledge structures, which in turn promotes deeper discussion and helps teams avoid premature consensus (Cronin et al., 2011; Mohammed et al., 2023; Srikanth et al., 2016). In this way, negative socio-emotional expressions may reflect more diverged rather than converged communication, contributing to richer team cognition (Mohammed et al., 2023). Thus,

*H3b*: Unequal AI access *increases negative* socio-emotional behaviors, which in turn influence cognitive diversity.

#### 2.3.2 Unequal AI access’ impact on task area interactions

In the IPA framework, task-related interactions are divided into questioning (e.g., asking for suggestions, opinions, or information) and answering behaviors (e.g., providing suggestions, opinions, or information) (Nam et al., 2009). Unequal AI integration creates information asymmetries and initial status expectation differences, as timely information and content-generation capabilities are more readily available to AI users. This results in concentrated questioning and answering behaviors, ultimately influencing cognitive diversity (Bunderson and Reagans, 2011).

First, non-AI users, facing information disadvantages, are more likely to seek orientation or advice from AI-equipped teammates to compensate for knowledge gaps. Simultaneously, AI users, seen as knowledge contributors, tend to take on the role of providing task-relevant input to facilitate team coordination (Rogers et al., 2009; Zhang et al., 2025). As a result, task-related communication—both questioning and answering—becomes increasingly concentrated. This interactional imbalance resulting from informational asymmetry can shape how information flows and integrates into teams, further affecting cognitive diversity. Specifically, such imbalanced information exchanges expose non-overlapping cognitive regions and stimulate cross-boundary information flow, which promotes knowledge integration (Bunderson and Sutcliffe, 2002; Mesmer-Magnus and DeChurch, 2009) and deepens analytical engagement (Homan et al., 2007). Ultimately, such patterns support the emergence of greater team cognitive diversity.

Moreover, access to AI may elevate expectations about one’s task contributions, making AI users often perceived as high-status actors within teams (Correll and Ridgeway, 2003). These status differences shape the direction of task-related communication (Bunderson and Reagans, 2011). For instance, higher-status members are more likely to assume directive roles by offering orientations and suggestions, whereas lower-status members tend to ask more questions and seek guidance from those perceived as more knowledgeable (Chung and Pennebaker, 2011; De Jong et al., 2022). Building on this dynamic, unequal AI access may initially create status-based expectations that result in questioning and answering behaviors becoming concentrated within specific individuals. Over time, such interaction patterns can reinforce and solidify team status hierarchies, which represent differentiated perspectives and styles in approaching tasks (Harrison and Klein, 2007). Thus, we propose the following hypotheses:

*H3c*: Unequal AI access increases concentrated task-related *questioning*, which in turn influences cognitive diversity.

*H3d*: Unequal AI access increases concentrated task-related *answering*, which in turn influences cognitive diversity.

**Figure 2:**
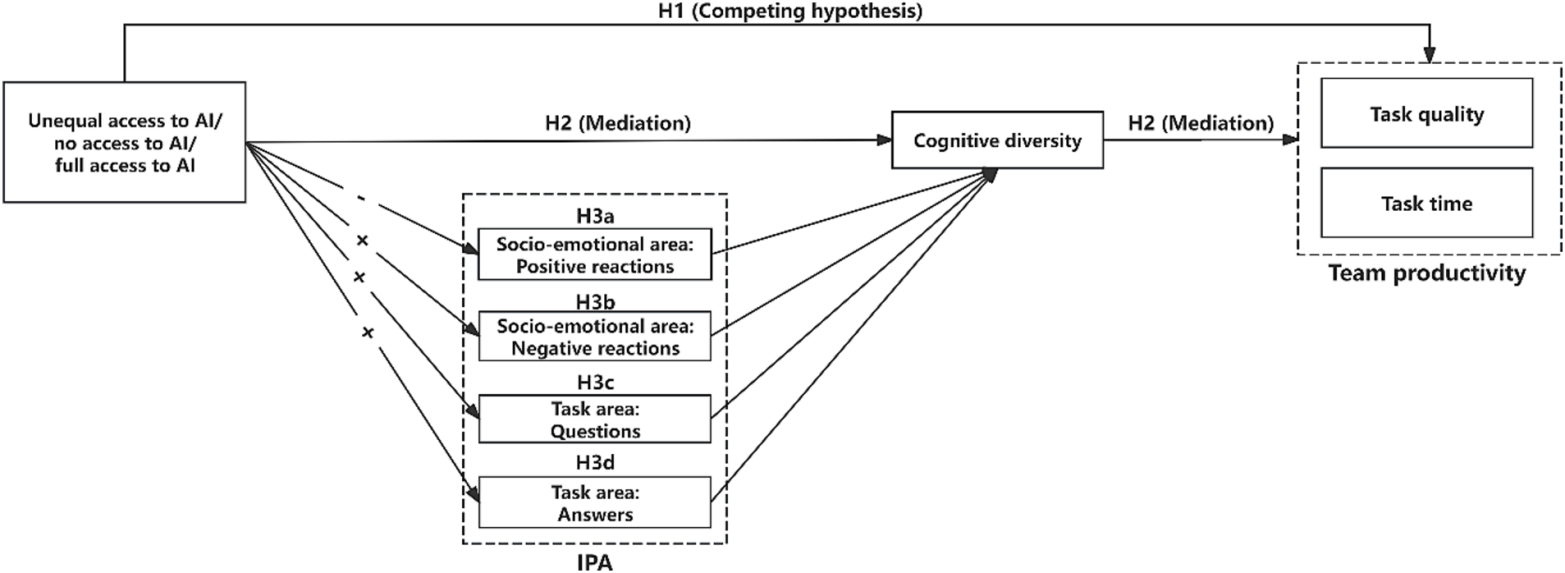
Research model.

The hypothesized research model, as depicted in Figure 2, integrates the serial mediation links between varied AI access structures, team interaction processes (further divided into socio-emotional and task-oriented processes), cognitive diversity, and team productivity (quality and time).

## 3 Method

We conducted a randomized and controlled laboratory experiment to examine how different AI integration structures influence team cognitive diversity and task performance through a press release writing task. The study recruited a total of 120 university students from various majors, who were randomly assigned to form 60 two-person teams. Each team first went through a control phase task where neither team member was permitted to use GenAI when completing the writing task (no access condition). Then, in the treatment phase, these teams were randomly assigned to one of two conditions: only one member could use GenAI (unequal access), or both members could use it (full access). For the GenAI tool, we employed Kimi 3.0, a Chinese-language-optimized large language model developed by Moonshot, chosen for its superior ability to handle lengthy text inputs and its suitability for the Chinese writing tasks.

### 3.1 Participants

This experiment involved 120 participants, who were made up of undergraduates and graduate students from a university in China. Our interest in studying face-to-face interactions in two-person teams made conducting the experiment with student samples the most feasible approach.

The sample of participants was 72% female, and a chi-square test of independence revealed that gender proportions did not significantly differ across the three experimental conditions, $\chi^{2}$ = (2, *N* = 120) = 0.082, *p* = 0.960. Approximately 18% of the sample were from the humanities and social sciences disciplines. Chi-square analysis indicated that the distribution of participants across the three conditions was statistically equivalent, $\chi^{2}$ = (2, *N* = 120) = 0.000, *p* = 0.999. Approximately 24% of the participants had prior experience related to marketing, with no significant differences observed across the three conditions, $\chi^{2}$ = (2, *N* = 120) = 1.455, *p* = 0.483. Approximately 95% of the participants had experience using generative AI tools (such as ChatGPT and Kimi), with no significant differences observed across the conditions, $\chi^{2}$ = (2, *N* = 120) = 0.790, *p* = 0.674. Participants received a reward of 50 RMB for participating in the experiment. Additionally, if their group’s overall task quality was rated above 6 (on a range of 1–7, 7 the highest), each task would earn an extra 10 RMB.

### 3.2 Experiment procedure

This study selected Kimi 3.0, a large language model (LLM) developed by the Chinese company Moonshot, as the generative AI tool for team use for two advantages. First, Kimi was trained in and optimized for the Chinese language, making it an ideal choice given the designed writing task in Chinese. Second, Kimi outperforms other large models available in China in its ability to handle long texts (Team et al., 2025). This allows Kimi to better comprehend participants’ extensive input commands and complete writing tasks more effectively.

**Figure 3:**
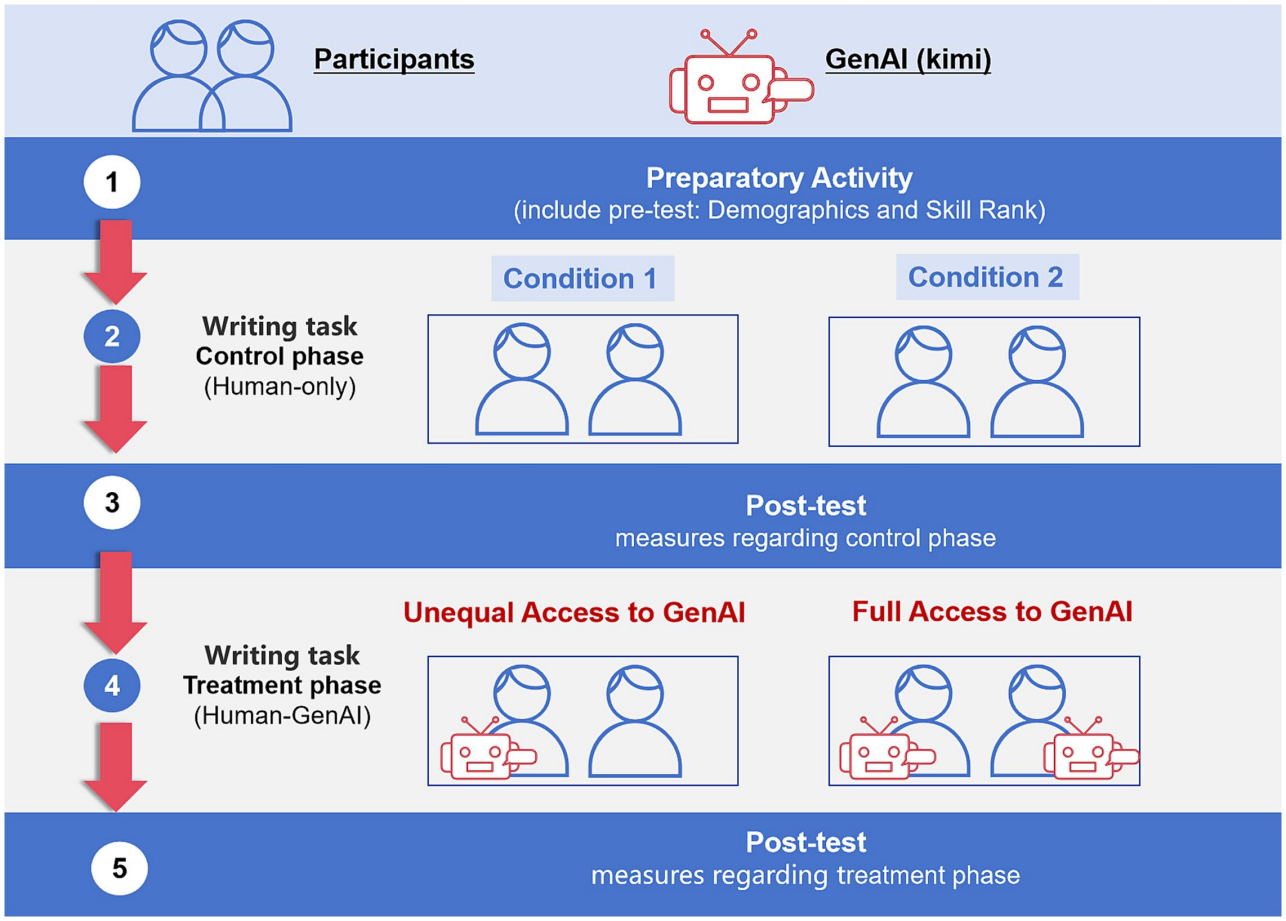
Experiment design.

Team activities were divided into five steps (shown in Figure 3): pre-test, control phase writing task, post-test 1, treatment phase writing task, and post-test 2. During the preparatory phase, participants completed an initial questionnaire to control for individual factors that could influence team communication and productivity, including demographic information, GenAI usage experience, and self-assessed skill levels in communication, creativity, and problem-solving. The first writing task served as the control task, where no members from any condition’s teams could use GenAI. The second writing task served as the treatment task, where in condition 1, only one of two members was randomly assigned access to GenAI, and in condition 2, both individuals could use GenAI to complete the writing task. The first condition represented teams with unequal access to GenAI, while the second condition represented teams with full access to GenAI. Team members always have access to computers configured with task instruction documents and basic document editing tools. Only the individuals allowed to use GenAI were provided with a link to Kimi, and there was no restriction on how to interact with Kimi. All participants were not allowed to use any other websites or applications when not instructed to do so. After the experiment, we reviewed the on-site recordings to ensure that each group carried out the tasks in accordance with the above-mentioned requirements. The experiment design was approved by the university IRB (H20240616I).

### 3.3 Writing task design

The entire experiment comprises two writing tasks, in which two-person teams were asked to collaboratively produce a 700-character press release about a hypothetical product (an electric bicycle in the control phase and an AR glasses product in the treatment phase). This writing task is adapted from team collaboration tasks designed in prior literature (Noy and Zhang, 2023). Each writing task should not exceed 45 min in duration. Before starting each task, every team was first given basic information about the hypothesized product and writing instructions (see the Supplementary material Section 1 for Writing Tasks Instructions).

### 3.4 Measures

#### 3.4.1 Access to GenAI

Access to GenAI serves as the main independent variable in the experiment. According to Hayes and Preacher (2014), we used indicator coding, also known as dummy coding, to represent this multi-categorical independent variable. To dummy-code three groups (no AI access, partial AI access, and full AI access), two dummy variables are constructed. The “No access” variable has a value of 1 if a case is in no access to the AI group and 0 otherwise. The “Full access” variable is set to 1 if a case is in the full AI access group and 0 otherwise. Partial AI access group functions as the reference category in the analysis and parameters reported in the model that are pertinent to group differences should be interpreted relative to this reference group.

#### 3.4.2 Team productivity

Team productivity in this study was assessed along two key dimensions: task quality and task time, reflecting both the effectiveness and efficiency of team output (Harrison et al., 2003; Noy and Zhang, 2023).

##### 3.4.2.1 Task quality

Following Noy and Zhang (2023), task quality was assessed by (blinded) expert raters working in marketing. Evaluators assigned an overall grade (1–7) to the writing task submissions based on three criteria: writing quality, content quality, and originality. Detailed instructions, including sample submissions with high and low scores, can be found in Section 2 in the Supplementary material. We recruited a total of nine professionals from the marketing industry as expert raters. Each of the 120 submissions was randomly assigned to three raters to ensure high reliability, with each rater evaluating 40 submissions. To encourage quality evaluations, raters were informed that their reward would be based on the correlation between their scores and those of the other raters. The Cronbach alpha between the three raters’ scores was 0.791.

##### 3.4.2.2 Task time

Task completion time was measured as the total duration each team spent working collaboratively on the assigned task. Following the procedures outlined by Noy and Zhang (2023), the entire task completion process was video-recorded for each team. Trained research assistants subsequently reviewed the recordings and extracted the task completion time for each team.

#### 3.4.3 Cognitive diversity

To obtain an objective measure of cognitive diversity within each team, we employed a computational text analysis approach developed by Gurkan and Yan (2023). Team discussions were first transcribed from audio recordings, with manual corrections to ensure accuracy. We then identified and concatenated each team member’s utterances across the entire team discussion. These text blocks were vectorized using the Universal Sentence Encoder (USE) from TensorFlow Hub (Cer et al., 2018), and each vector was normalized such that its magnitude (Euclidean norm) equals 1. Cognitive diversity was then calculated as the cosine distance (*d*) between the normalized vectors of the two team members (*i* and *j*), with higher values of *d* indicating greater cognitive dissimilarity. That is,


$$d(W_{i},W_{j})=1-\cos(W_{i},W_{j})$$

where $W_{i}$ denotes the concatenated spoken text expressed by the individual *i.*

#### 3.4.4 Team interaction process

Team interaction behaviors were coded using Bales’ Interaction Process Analysis (IPA) framework, which includes 12 subcategories grouped into four functional areas: positive socio-emotional, negative socio-emotional, task-related answering, and task-related questioning (see Section 3 in the Supplementary material).

The unit of analysis was a single simple sentence or its equivalent—the smallest independent unit of meaning (Bales, 1950). Coders were instructed to treat short, complete responses (e.g., “Yes,” “I agree”) as standalone units. In contrast, sentence fragments that depend on preceding or following speech (e.g., “Because.,” “And then.”) should be merged with the adjacent utterance. Additionally, coders were trained to avoid combining sequential but distinct behaviors into a single code. For example, the utterance ‘Yes, that makes sense, and what should we do next?’ should be coded as two separate units—one for *Agreement* and one for *Asks for Suggestions*.

After the initial training, two coders independently coded 25% of the data. Discrepancies in this subset were discussed and resolved to refine the coding scheme for clarity. Once the coders achieved satisfactory agreement, they completed the remaining dataset. The final results yielded a Cohen’s Kappa of 0.77, indicating substantial reliability. Any remaining disagreements were resolved through discussion, and consensus codes were used for analysis.

##### 3.4.4.1 Socio-emotional area reactions

For each task group, the socio-emotional area behaviors were computed as relative frequency scores for the positive and negative interaction behaviors:

Positive socio-emotional behavior = Positive units/Total communication units.

Negative socio-emotional behavior = Negative units/Total communication units.

##### 3.4.4.2 Task area reactions

For each task group, the task-related reactions were calculated as the ratio between two members’ questioning or answering behaviors. Specifically, the ratio was determined by dividing the higher count of questioning behaviors by the lower count for each pair of team members. The formula used is as follows:

Concentrated questioning = max (Questioning units by A, Questioning units by B)/ min (Questioning units by A, Questioning units by B).

Concentrated answering = max (Answering units by A, Answering units by B)/ min (Answering units by A, Answering units by B).

Larger ratio scores indicate a higher level of concentration, meaning one member dominated that specific behavior (e.g., questioning or answering) to a greater extent. There exists a great imbalance between the two members in Q&A behaviors.

#### 3.4.5 Control variables

We aggregated demographic variables to the team level, resulting in three control variables:

**Female proportion**. Proportion of female members in each team, calculated as the number of females divided by total team size (e.g., 0, 0.5, or 1 in two-person teams).

**Marketing experience**. If at least one member of a team has education or working experience in marketing-related education or work, this variable is marked as 1; if not, it is marked as 0.

**Team skill**. Participants were asked to rank their level in the following three teamwork skills: being an effective communicator, being creative and original, and problem-solving (Noy and Zhang, 2023). Each participant assigned a score of 3 to the skill they ranked first, 2 to the second, and 1 to the third. Based on these individual scores, we calculated team-level scores for each skill by averaging across team members, resulting in three variables: *team communication ability*, *team problem-solving ability*, and *team creativity*.

Due to concerns of multicollinearity (as the three scores are interdependent and sum to a constant), we included only *problem-solving skill* and *creativity* as control variables in our main analyses.

## 4 Results

To investigate how unequal access to AI predicts team task quality and completion time through team interaction processes and cognitive diversity, we conducted a PROCESS macro analysis. In this model, AI access (unequal access/ no access/ full access) served as the multi-categorical independent variable (IV); the four types of team interaction and cognitive diversity were included as mediators; and team productivity—task quality and task time—were treated as the dependent variables (DVs). We set the unequal AI access condition as the reference group and compared it with the no AI access condition (X₁) and the full AI access condition (X₂).

We first tested the hypothesized model, which demonstrated a good fit to the data: χ^2^ (6, *N* = 60) = 4.243, *p* = 0.644. The probability that the root mean square error of approximation (RMSEA) is less than or equal to 0.05 was 0.900, and the other fit indices also indicated excellent model fit: comparative fit index (CFI) = 1.000, Tucker-Lewis index (TLI) = 1.027, and standardized root mean square residual (SRMR) = 0.0019. Having confirmed the overall model fit, we proceeded to examine each path in the model to evaluate our hypotheses.

### 4.1 Unequal access to GenAI leads to higher task quality and faster task completion

**Figure 4:**
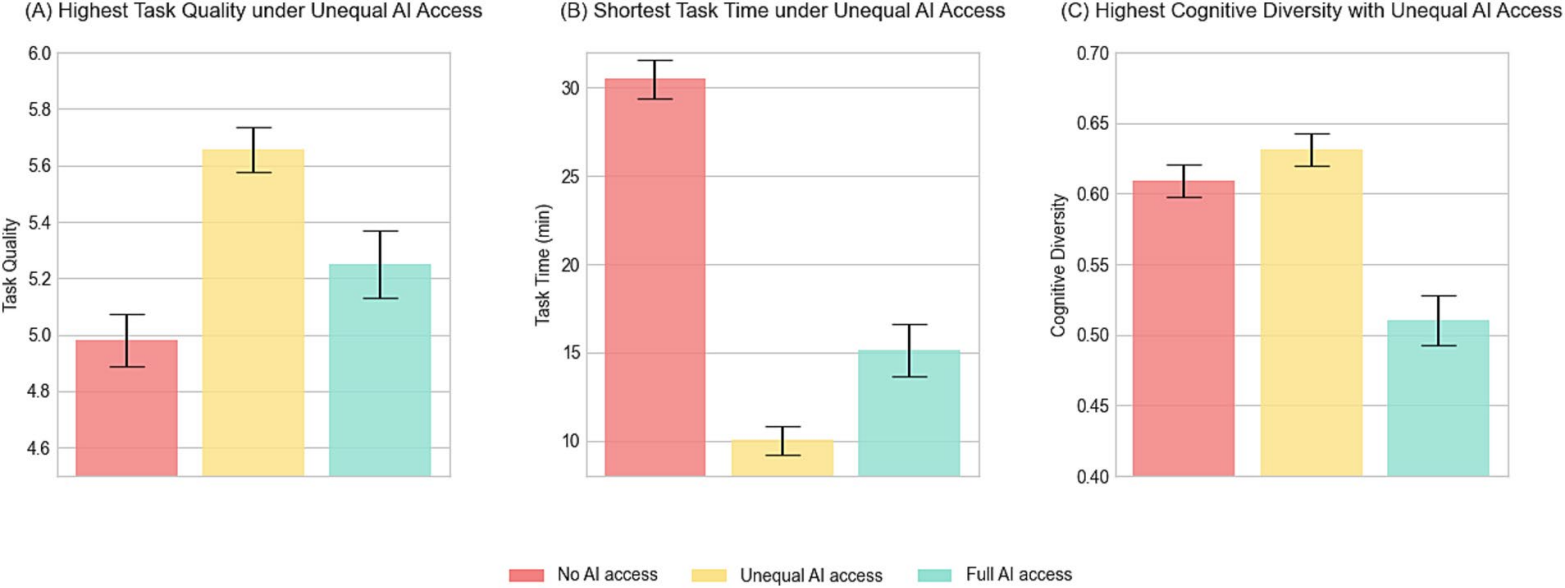
Team outcomes under different AI access conditions. Bar charts depict **(A)** task quality, **(B)** task completion time, and **(C)** cognitive diversity under three AI access conditions: no AI access, unequal AI access, and full AI access. Error bars reflect ±1 standard error of the mean.

To examine the overall effect of AI integration structure on team productivity (H1), we compared task quality and completion time across the three AI integration conditions. As illustrated in Figure 4A, task quality in teams with unequal AI access (*M* = 5.654, *SD* = 0.080) was higher than in teams with no AI access (*M* = 4.981, *SD* = 0.094), *t* = −5.103, *p* < 0.01, and those with full AI access (*M* = 5.250, *SD* = 0.118), *t* = 2.881, *p* < 0.01. This finding is further supported by the OLS results reported in Model (1) of Table 1, where unequal AI access was associated with higher task quality compared to both the no access condition (*b* = −0.673, *p* < 0.01) and the full access condition (*b* = −0.402, *p* < 0.05).

**Table 1:** Regression of different AI access on team productivity.

Variable	(1) Task quality		(2) Task time	
	*b*	*SD*	*b*	*SD*
No access to AI	−0.6730^***^	−5.1990	20.7523^***^	14.1546
Full access to AI	−0.4023^**^	−2.2771	5.9820^***^	2.9896
Female proportion	−0.0123	−0.0593	1.3377	0.5705
Team problem-solving ability	0.0624	0.3415	−1.9904	−0.9619
Team creativity	0.2113	1.4935	−2.1255	−1.3264
Marketing experience	−0.0176	−0.1357	3.2350^**^	2.2036
Constant	5.1217^***^	10.1856	15.6672^***^	2.7509
*N*	120		120	
adj. *R*^2^	0.170		0.644	

*t* statistics in parentheses. ^*^p < 0.1, ^**^p < 0.05, ^***^p < 0.01.

A similar trend was observed in task completion time (Figure 4B). Teams with unequal AI access completed the task faster (*M* = 10.013, *SD* = 0.807) than human-only teams (*M* = 30.483, *SD* = 1.108; *t* = 13.556, *p* < 0.01), and those with full AI access (*M* = 15.150, *SD* = 1.491; *t* = −3.312, *p* < 0.01). These time savings are further reflected in the OLS estimates reported in Model (2) of Table 1, which show significantly reduced task duration for the unequal access condition compared to both no AI (*b* = 20.752, *p* < 0.01) and full AI access (*b* = 5.982, *p* < 0.01). Therefore, these findings provide support for H1a, indicating that unequal access to AI can significantly enhance team productivity by improving task quality and accelerating task completion.

### 4.2 Cognitive diversity links unequal AI access with enhanced task quality

**Figure 5:**
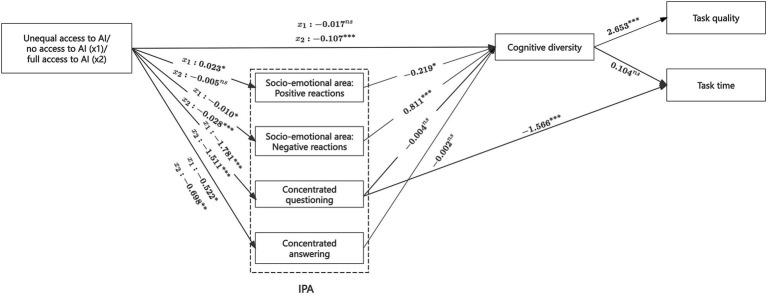
Results of the mediation model (with unequal access condition as reference group). ***p < 0.01, **p < 0.05, *p < 0.1, ns, not significant. Other non-significant paths are omitted in the figure.

To evaluate the hypothesized mediating role of cognitive diversity (H2), we first tested whether AI integration structure significantly influences cognitive diversity and whether cognitive diversity, in turn, predicts team productivity. Independent-sample *t*-tests were conducted to compare communication responses across three different AI access conditions, serving as a proxy for cognitive diversity. As shown in Figure 4C, teams with unequal AI access exhibited significantly higher cognitive diversity (*M* = 0.631, *SD* = 0.011) than those with full AI access (*M* = 0.510, *SD* = 0.018), *t* = 5.938, *p* < 0.01. However, no significant difference was observed between the unequal AI access group and the no AI access group (*M* = 0.609, *SD* = 0.012), *t* = −1.299, *p* = 0.197. Further results from the mediation model (Figure 5) supported the pattern observed in the above findings. Compared to teams with unequal AI access (reference group), those with full AI access showed significantly lower cognitive diversity (*b* = −0.107, *SE* = 0.025, *p* < 0.01), while human-only teams did not differ significantly (*b* = −0.017, *SE* = 0.020, *p* > 0.1).

Importantly, the PROCESS model confirmed that cognitive diversity was positively associated with task quality (*b* = 2.653, *SE* = 0.752, *p* < 0.01), but showed no significant effect on task completion time (*b* = 0.104, *SE* = 7.454, *p* > 0.1). Bootstrapped indirect effect analysis (Table 2) further validated the mediating role of cognitive diversity: the indirect effect of unequal AI access (vs. full AI access) on task quality via cognitive diversity was significant (*b* = −0.283, *SE* = 0.091, 95% CI [−0.513, −0.134]). This suggests that unequal AI access can enhance team effectiveness by fostering greater cognitive diversity. In sum, these findings support H2 by demonstrating that cognitive diversity significantly mediates the relationship between AI integration structure and team productivity—specifically, by enhancing task quality.

**Table 2:** Significant indirect effects tested by 95% bootstrapped confidence intervals.

Indirect path	Estimated effect	95%CI
Unequal AI access (versus full AI access) → cognitive diversity → task quality	−0.283	[−0.513, −0.134]
Unequal AI access (versus full AI access) → negative socio-emotional reactions → cognitive diversity → task quality	−0.061	[−0.148, −0.021]
Unequal AI access (versus no AI access) → concentrated task-related questioning → task time	2.790	[1.628, 4.138]
Unequal AI access (versus full AI access) → concentrated task-related questioning → task time	2.367	[0.780, 4.578]

### 4.3 Mechanisms underlying the impact of GenAI access on cognitive diversity and team productivity

#### 4.3.1 Socio-emotional area: negative reactions and cognitive diversity act as serial mediators between unequal AI access and task quality

H3a and H3b proposed that socio-emotional team interactions—positive and negative reactions—mediate the relationship between AI integration structure and cognitive diversity. Path analyses revealed that unequal AI access significantly increased negative socio-emotional behaviors compared to full AI access (*b* = −0.028, *SE* = 0.007, *p* < 0.01), which, in turn, positively influenced cognitive diversity (*b* = 0.811, *SE* = 0.242, *p* < 0.01), supporting H3b. However, no significant effects were found for AI integration structure on positive reactions (relative to no access: *b* = 0.023, *SE* = 0.014, *p* > 0.05; relative to full access: *b* = −0.005, *SE* = 0.017, *p* > 0.05), thus failing to support H3a.

As previously demonstrated, cognitive diversity mediates the relationship between unequal AI access and task quality. Building on this, we further tested whether negative socio-emotional interactions contribute to this indirect pathway. Results from the PROCESS model (Table 2) showed a significant bootstrapped serial indirect effect involving AI integration, negative socio-emotional behaviors, cognitive diversity, and task quality (relative to full AI access: *b* = −0.061, *SE* = 0.029, 95% CI [−0.148, −0.021]). These findings suggest that unequal AI access can enhance task quality by increasing negative socio-emotional reactions, which in turn promote greater cognitive diversity. In other words, negative interpersonal communication and cognitive diversity function as sequential mediators linking unequal AI access to improved team productivity.

#### 4.3.2 Task area: concentrated questioning mediates the relationship between unequal AI access and task time

To test the role of task-related interactions, H4a and H4b focused on whether concentrated questioning and answering mediate the link between AI integration and cognitive diversity. Path analysis (Figure 5) indicated that unequal AI access significantly increased both concentrated questioning (relative to no access: *b* = −1.781, *SE* = 0.381, *p* < 0.01; relative to full access: *b* = −1.511, *SE* = 0.452, *p* < 0.01) and concentrated answering behaviors (relative to no access: *b* = −0.522, *SE* = 0.278, *p* < 0.1; relative to full access: *b* = −0.698, *SE* = 0.295, *p* < 0.05). While the direction of these relationships aligned with our assumptions, the mediating effects did not. Concentrated task-related questioning (*b* = −0.004, *SE* = 0.005, *p* > 0.1) and answering behavior (*b* = −0.002, *SE* = 0.007, *p* > 0.1) showed no significant impact on cognitive diversity, contrary to H3c and H3d.

Although task-related behaviors did not mediate the relationship between AI integration and cognitive diversity, we found that concentrated questioning had a direct negative effect on task time (*b* = −1.566, *SE* = 0.502, *p* < 0.01). The bootstrapped indirect effects (Table 2) from unequal AI access to task time through concentrated questioning were significant (relative to no access: *b* = 2.790, *SE* = 0.658, 95% CI [1.628, 4.138]; relative to full access: *b* = 2.367, *SE* = 0.702, 95% CI [0.780, 4.578]). These findings suggest that unequal AI access can shorten task time by prompting non-AI users to take on a greater share of task-related questioning, thereby increasing the efficiency of team interactions.

### 4.4 Robustness check

This study presents two additional analyses to strengthen the robustness of the findings reported above.

#### 4.4.1 Baseline team characteristics comparison

To ensure that there are no significant differences in team baseline characteristics across conditions and to rule out the impact of initial levels on the observed outcomes, *t*-tests were conducted on various team characteristics. Table 3 provides descriptive statistics for team-level control variables, task quality, task time, cognitive diversity, and four categories of team interaction processes during the control phase. *t* tests compared these variables across two conditions and found no significant differences in terms of baseline team characteristics.

**Table 3:** Descriptive statistics.

Variable	Condition 1 (unequal access)	Condition 2 (Full access)	*t* tests
	(*N* = 40)	(*N* = 20)	
	Mean (*SD*)	Mean (*SD*)	*t* (*p*)
Female proportion	0.725 (0.054)	0.700 (0.076)	0.269 (0.789)
Marketing experience	0.400 (0.496)	0.200 (0.410)	1.555 (0.126)
Team problem-solving ability	1.763 (0.059)	1.925 (0.098)	−1.495 (0.140)
Team creativity	2.075 (0.085)	2.000 (0.115)	0.517 (0.607)
Task quality	4.958 (0.818)	5.025 (0.508)	−0.333 (0.740)
Task time	30.550 (9.419)	30.350 (6.831)	0.084 (0.933)
Cognitive diversity	0.618 (0.102)	0.591 (0.063)	1.103 (0.274)
Positive socio-emotional reactions	0.142 (0.058)	0.155 (0.060)	−0.866 (0.390)
Negative socio-emotional reactions	0.027 (0.028)	0.025 (0.024)	0.268 (0.790)
Concentrated task-related questioning	1.347 (0.056)	1.297 (0.055)	0.560 (0.578)
Concentrated task-related answering	2.186 (0.205)	1.781 (0.230)	1.217 (0.229)

#### 4.4.2 Measuring task quality by originality

In the main analysis, task quality was assessed through three dimensions—content quality, writing quality, and originality (see section 3.4.2). Given that cognitive diversity is widely acknowledged to influence team creativity (Mathuki and Zhang, 2024; Qi et al., 2022; Wang et al., 2016), we identified originality as the core dimension most directly driven by cognitive diversity. To test the robustness of our findings, we re-ran the mediation analysis using originality as the sole indicator of task quality.

**Figure 6:**
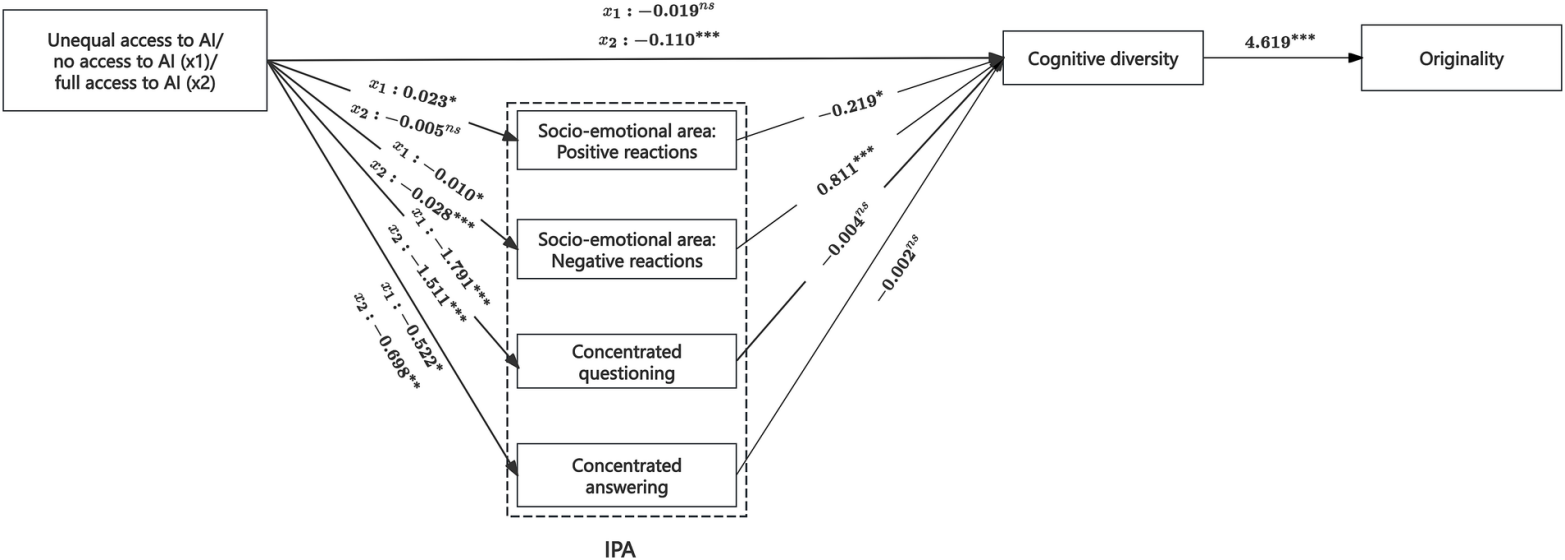
Results of the mediation model (with task quality measured by originality). ***p < 0.01, **p < 0.05, *p < 0.1, ns, not significant. Paths related to task time are omitted for clarity.

The model exhibited a good fit (χ^2^ (6, *N* = 60) = 2.243, *p* = 0.644). As shown in Figure 6, cognitive diversity had a significant positive effect on originality (*b* = 4.619, *SE* = 1.346, *p* < 0.01). Moreover, the serial mediation pathway from unequal AI access to originality—via negative socio-emotional interactions and cognitive diversity—was also significant, relative to full AI access (*b* = −0.105, *SE* = 0.050, 95% CI [−0.248, −0.036]). These results provide robust support for our earlier conclusions, reaffirming that unequal access to GenAI enhances task quality primarily through its effect on team interaction dynamics and cognitive diversity, particularly as reflected in originality.

#### 4.4.3 Measuring concentrated task-related behavior using difference scores

To test whether our findings are sensitive to how Concentrated Task-Related Behavior is measured, we re-estimated the model using an alternative operationalization based on difference scores (De Jong et al., 2022).

Concentrated questioning = |Questioning units by A – Questioning units by B| / Total questioning units.

Concentrated answering = |Answering units by A – Answering units by B| / Total Answering units.

A and B represent the two team members. Higher values indicate a greater concentration of the corresponding behavior within teams.

**Figure 7:**
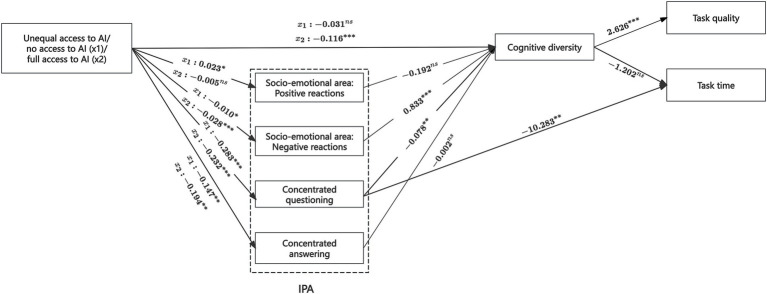
Results of the mediation model (with concentrated task-related behavior measured using difference scores). ***p < 0.01, **p < 0.05, *p < 0.1, ns, not significant. Other non-significant paths are omitted in the figure.

The results (Figure 7) show that the overall model fit remained acceptable under this alternative specification (χ^2^ (6, *N* = 60) = 5.752, *p* = 0.452). Importantly, the hypothesized indirect path from unequal AI access to task time via concentrated questioning behavior remained statistically significant (relative to no access: *b* = 2.913, *SE* = 1.138, 95% CI [1.003, 5.399]; relative to full access: *b* = 2.390, *SE* = 1.059, 95% CI [0.778, 4.939]), supporting the robustness of the proposed mechanism.

## 5 Discussion

### 5.1 Key findings

Our analysis revealed four key patterns. First, contrary to a general intuition that fully equipping working teams with GenAI could enhance team productivity, we observe that teams with unequal AI access actually improved task quality by improving team cognitive diversity. Though unequal AI access does not seem to affect task time. Second, when examining team interactions, unequal AI access also had some interesting effects. In the socio-emotional area interactions, it sparked more negative reactions, like disagreement, but did not really change how often people expressed positive emotions. In the task area interactions, it led to more concentrated task-related questioning and answering, with certain team members taking the lead in asking questions and others concentrating on answering them. Third, more concentrated task-related questioning explains why unequal AI access (versus full and no access) reduced task time. That is, when a subset of team members primarily handles questioning, task completion accelerates. Fourth, there exists a positive serial mediation path from unequal AI access (versus full access) to improved task quality, sequentially through increased negative socio-emotional behaviors and greater cognitive diversity. In other words, although unequal access led to more disagreement among team members, this also encouraged a broader range of thinking styles—ultimately helping the team perform better.

### 5.2 Theoretical implications

This study offers three key theoretical contributions to the literature on AI integration structures and team processes in HATs. First, we clarify conflicting perspectives on the relationship between unequal AI access and team productivity through the lens of cognitive diversity. We find that cognitive diversity induced by partial AI access enhances task quality, aligning with previous findings by Wang et al. (2016) and Aggarwal and Woolley (2019), which emphasize the value of diverse perspectives in team collaboration. In terms of task completion time, cognitive diversity has no significant impact, in contrast to prior literature that documented both its positive (Li et al., 2022; Pieterse et al., 2011) and negative (Mohammed and Schillinger, 2022; Narayan et al., 2021) effect on team working efficiency. Thus, we emphasize the role of cognitive diversity as a key mediator through which unequal AI access improves the quality of creative task outputs.

Second, by building and testing an I-P-S-O model, we theorize and empirically demonstrate that unequal AI access gives rise to distinctive interaction processes (P factor) and emergent cognitive states (S factor), which sequentially mediate its impact on teaming effectiveness. This contributes to team science literature by identifying the underlying mechanisms through which inconsistent technological usage shapes collaborative dynamics. Moving beyond the view of AI as a uniform group-level resource (e.g., Gurkan and Yan, 2023), we demonstrate how individual-level differences in AI technology access may actively reshape information distribution within teams. Specifically, we find that unequal AI access alters the flow of communication by concentrating questioning and answering behaviors within certain members. In other words, when AI access is unequal, information flows become more fixed: some members possess more task-relevant information and thus predominantly answer questions, while others, lacking such information, primarily ask questions. This pattern corresponds to the I-P path of our I-P-S-O model. Furthermore, informational asymmetry caused by unequal AI integration fosters deeper discussions, thereby enhancing team cognitive diversity. This reflects the I-S path in our model. Our findings show that unequal access stimulates more diverse perspectives, whereas full access may have a homogenizing effect by leading team members to base their reasoning and decisions on similar AI-generated inputs. This pattern aligns with prior research on social confirmation bias (Lu et al., 2012; Stasser and Titus, 2003), which shows that shared information among members can overshadow unique contributions and suppress cognitive diversity.

Moreover, this study finds that cognitive diversity can emerge dynamically from team interaction processes, which refers to the P-S link in the IPSO model. We empirically identify negative socio-emotional behaviors, especially disagreement, in team communication that are most strongly associated with the emergence of cognitive diversity. This finding supports theoretical propositions by Marks et al. (2001) and Mello and Rentsch (2015), who suggested that cognitive diversity functions as an emergent property shaped by ongoing team dynamics. Our study thus offers empirical insight into the interpersonal communicative mechanisms underpinning the development of cognitive diversity in human-AI teams.

Third, our study also explores the direct effects of varied aspects of team interaction dynamics on team productivity. Unlike prior studies that treat interactions as a general concept (Gurkan and Yan, 2023; Mello and Rentsch, 2015), we differentiate interactions in the task and socio-emotional domains and find that they each have distinct effects on task completion time and task quality, respectively. In the task domain, team interactions characterized by concentrated patterns of questioning are closely associated with shorter task completion times. These patterns only arise when the GenAI access is partial, meaning that AI users may tend to provide orientation and information, while non-users seek suggestions and ask more questions. Interestingly, only concentrated task-related questioning—rather than answering—appears to accelerate task completion. This may be explained by De Jong et al. (2022), who argue that questioning can signal recognition of others’ expertise or leadership, suggesting that AI access may function as a status characteristic, reinforcing status hierarchies and improving decision-making efficiency. Both theoretical explanations offer interesting insights worthy of future empirical testing.

In the socio-emotional domain, negative interactions—particularly those stemming from disagreement under conditions of unequal AI access—are found to have a positive impact on task quality. While this finding partially aligns with prior research (Mesmer-Magnus and DeChurch, 2009; Stasser and Titus, 1985; Van Knippenberg and Schippers, 2007), which highlights that uneven information distribution can lead to conflict, those studies typically view such conflict as detrimental to team cohesion and performance. In contrast, our findings suggest that task-related disagreement, though seemingly negative, may stimulate deeper cognitive engagement and enhance team outcomes. This supports the view of Farh et al. (2010), who argue that moderate task conflict can benefit collaboration and creativity.

In conclusion, our IPSO model proposes a comprehensive influence pathway—from AI integration structure as a team input, through observable team interaction behaviors and cognitive emergent states, to team productivity such as task quality and completion time. This enriches the IMO model for HATs proposed by O'Neill et al. (2023), providing a theoretically grounded explanation of how varying levels of GenAI access shape emergent cognition and collaborative performance. Our findings offer a foundation for developing strategic GenAI integration frameworks to optimize human-agent collaboration in diverse team environments.

### 5.3 Practical implications

In practical terms, this paper provides assistance and guidance for establishing management strategies for short-term Human-GenAI teams. The two-person teams in this study can be expanded to multi-person teams in the real world. We demonstrate that unequal GenAI access among team members can reshape information flows and influence team cognitive diversity, thereby impacting task quality. Rather than simply pursuing equal access across all members, organizations should consider the strategic allocation of GenAI based on task requirements, member roles, and the desired level of cognitive diversity. For instance, in short-term collaborative tasks that require innovative problem-solving—such as brainstorming sessions or team debates—a certain level of cognitive divergence resulting from differentiated AI usage may be beneficial. However, for teams that emphasize long-term relationships and the personal development of members, alternative allocation strategies may be more appropriate. By strategically limiting access to GenAI, organizations can potentially harness the strengths of both human expertise and AI capabilities, fostering an environment in which diverse perspectives contribute to both task outcomes and team development.

In light of our findings, team leaders and facilitators should actively monitor and manage interaction patterns that emerge from unequal GenAI integration. Our results suggest that disagreement stemming from unequal AI distribution is not inherently detrimental; in fact, it significantly enhances team cognitive diversity, which in turn improves the quality of team output. Therefore, when task-related disagreements arise between AI users and non-users, managers need not suppress such conflict. Instead, they should view it as a potential catalyst for creativity, intervening only to guide it constructively. However, when such task conflict escalates into relationship conflict or fosters mistrust among members, targeted interventions become necessary to maintain psychological safety and team cohesion.

### 5.4 Limitations and future directions

While our study provides valuable insights into team cognition states under varied AI integration structures, several limitations should be acknowledged to inform future research and deepen understanding of the topic. First, our sample was somewhat limited in its diversity due to practical constraints in recruiting participants for a controlled laboratory experiment involving face-to-face team interactions. Recruiting student participants was the most feasible and appropriate approach given resource availability and the need for experimental control. While the student sample included individuals from a broad range of academic disciplines, reflecting some diversity in cognitive and educational backgrounds, it is important to note that these participants generally lack substantial real-world work experience and exposure to professional team environments. This limitation may affect the external validity of our findings.

Second, there was a notable gender imbalance in our sample. Prior research suggests gender can influence perceptions of status (Levin, 2004; Ridgeway, 2001), communication style (Furumo and Pearson, 2007), and participation equity within teams (Bear and Woolley, 2011). Although we conducted additional post-hoc analyses and found no significant gender differences in team processes or productivity across different AI access conditions, this issue warrants further investigation. Future research should seek more gender-balanced samples to ensure robustness and broader applicability of the findings.

Third, the design of the team task has certain constraints. In this study, participants were required to complete the task through real-time communication within a limited amount of time, a format that mirrors many real-world settings, such as problem-solving meetings or short-term team competitions. However, the impact of AI integration structures in long-term collaboration remains an important area of exploration. In extended projects, task roles tend to be more clearly defined, and learning processes become more prominent. Emotional connections among team members may also deepen. Whether unequal AI access continues to outperform full AI access in fostering cognitive development in such contexts is a question worth investigating. Additionally, the control and treatment tasks used different product prompts—an electric bicycle and AR glasses, respectively. Although both prompts were pre-tested by domain experts to ensure similar levels of difficulty, complexity, and creative demand, this variation may still introduce uncontrolled differences in team performance. This design decision aimed to reduce learning and fatigue effects from task repetition, but future research would benefit from employing counterbalanced or equivalent task designs to further validate the robustness of the findings.

Finally, our study used a text-to-text interaction modality when prompting AI. Although this is currently the most mainstream interaction modality, future team collaboration may involve multimodal interactions, such as voice-based communication. It remains an open question whether multimodal interfaces could reduce the asymmetry in information and perceived status brought about by unequal AI access, thereby influencing interaction behaviors and cognitive states differently. This presents a promising direction for future research.

## 6 Conclusion

Team cognitive emergent states have long been recognized as critical components of team processes. This study explores how varied GenAI integration structures within HATs influence team cognitive diversity and, in turn, affect team productivity in areas such as task quality and efficiency. By uncovering the behavioral mechanisms—such as disagreement—that link AI access to divergent communication, this research deepens the understanding of how cognitive diversity emerges under unequal AI access. These differences in team cognition significantly enhance team output quality. Overall, this study highlights the central role of interaction dynamics and cognitive diversity in shaping team outcomes under varying patterns of GenAI use. Future work should continue to examine the nuanced mechanisms and interaction mode behind GenAI integration to better support collaborative performance in increasingly hybrid human-AI environments.

## Data Availability

The raw data supporting the conclusions of this article will be made available by the authors, without undue reservation.
