# Supplementary material for: Why unequal AI access enhances team productivity: the mediating role of interaction processes and cognitive diversity
Source: Front Psychol. 2025 Sep 8;16:1636906. doi: 10.3389/fpsyg.2025.1636906 (PMC12450962; doi:10.3389/fpsyg.2025.1636906)
Supplement: Supplementary file 1 [file Table_1.DOCX]

Supplementary Material

# Writing Tasks

**Instruction of Writing Task in Control Phase**

Write a 400-word press release for the following product: a self-driving electric bicycle called the *SmartBike*. Your goal is to maximize how attention-grabbing and attractive to potential consumers the press release is. The raters will grade your press release on this basis. Please keep in mind the following facts:

- The bicycle is being produced by a company, E-Bike Inc, that prides itself on the convenience and environmental friendliness of its products. Its customer base consists of mostly middle-class city-dwellers.
- E-Bike Inc is hoping to market the *SmartBike* to three populations: existing bicycle users, people who currently commute by walking, and people who currently commute by car. Your press release should discuss the advantages of the *SmartBike* relative to all three of these alternative commuting methods.
- In focus groups, many participants expressed concerns about the safety of a self-driving bicycle. Make sure to address these concerns in your press release by referencing the bicycle's spotless track record in testing and E-Bike Inc's care for the safety of its customers.
- The *SmartBike* has the following features:
- Up to 60 hours of battery life.
- Extremely advanced obstacle navigation, speed control, and braking systems.
- An integrated light system to signal turns and lane changes to drivers and enhance visibility in the dark.
- A lightweight and strong carbon-fiber frame. Integration with smartphones and watches.
- A remote-control mode that allows users to, for example, summon the bike from where it is parked.

Image of the *SmartBike*:


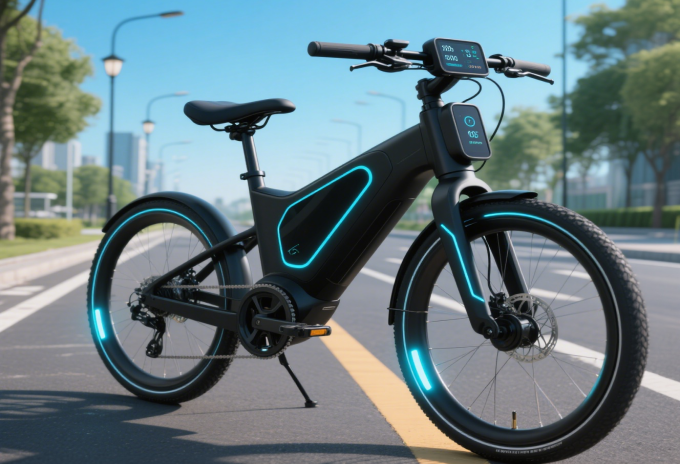


**Note:** The text for the *Instruction of Writing Task in Control Phase* is adapted based on the supplementary materials of Noy & Zhang (2023, p.69). The accompanying figure was created by the first author.

**Instruction of Writing Task in** **Treatment Phase**

Write a 400-word press release for the following product: *augmented-reality glasses* that allow a user to overlay digital images onto the real world. Your goal is to maximize how attention-grabbing and attractive to potential consumers the press release is. The raters will grade your press release on this basis.

Please keep in mind the following facts:

- The glasses are being produced by a tech startup called VAR lnc, whose first product was the *GameSet*, a very popular and successful virtual-reality headset for video gaming. The press release should include a callback to the *GameSet*, so that consumers associate the glasses with the highly popular *GameSet*.
- VAR Inc want to focus on two main uses of the glasses: enhancing productivity at work or in business settings, and fun recreational usage. The press release should include discussion and examples of both uses.
- Previous augmented-reality glasses have been commercially unsuccessful because users felt they were uncool or nerdy and were reluctant to wear them. The press release should try to make the glasses seem cool and trendy.

lmage of the augmented reality glasses:


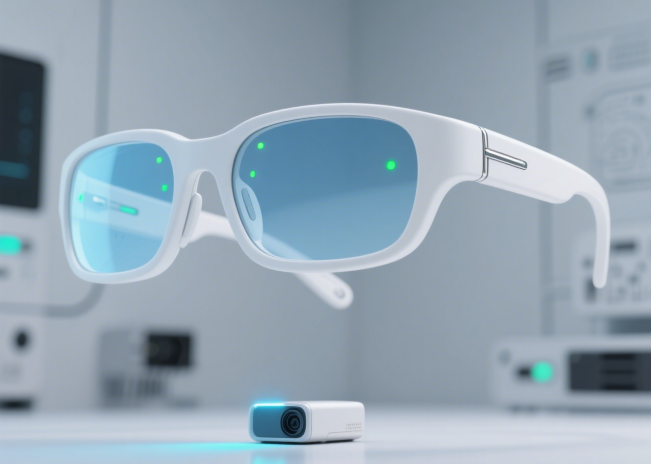


**Note:** The text for the *Instruction of Writing Task in Treatment Phase* is adapted based on the supplementary materials of Noy & Zhang (2023, p.72). The accompanying figure was created by the first author.

# Task Quality Scoring Criteria

Below are the scoring criteria for team performance (Noy & Zhang, 2023):

**Writing quality**

- Is the text well-structured and easy to follow?
- Are the paragraphs and sentences logically organized?
- Is the language clear and easy to understand?
- Are technical terms and jargon explained or avoided?
- Is the text free of errors in grammar, punctuation, and spelling?

**Content quality**

- Does the text effectively answer the prompt provided?
- Is the information accurate and comprehensive?
- Would the intended audience be receptive to the text?
- Is the text engaging?

**Originality**

- Are the ideas presented original and creative?

# Bales’ Interaction Process Analysis Categories with Examples

**Table 1. Bales’ Interaction Process Analysis (IPA) Categories and Examples**

| Functional Area | Category | Examples from our dataset |
| --- | --- | --- |
| Socio-emotional area: Positive  reactions | 1. Shows Solidarity/Seems Friendly: Any act that shows positive feelings toward another person | You’re amazing! |
|  | 2. Shows Tension Release/Dramatizes: Any act that reduces the anxiety that a person or group may be experiencing | I think we’ve done really well! |
|  | 3. Shows Agreement: Any act that shows acceptance of what another person has said | OK, I agree with you. |
| Task area: Attempted answers | 4. Gives Suggestions: Any act that offers direction/action for how to engage the task | Let's make the changes based on AI. |
|  | 5. Gives Opinions: Any act that advances a belief or value that is relevant to the task | I think its ability to summarize might be a bit weaker. |
|  | 6. Gives Orientation/Information: Any act that reports factual observations or experiences | It generated around 700 words. |
| Task area: Questions | 7. Asks for Orientation/Information: Any act that requests factual observations or experiences | Are you trying the instructions they gave? |
|  | 8. Asks for Opinions: Any act that requires a belief or value that is relevant to the task | How do you think it wrote? |
|  | 9. Asks for Suggestions: Any act that requests direction/action for how to engage the task | How should we divide the work? |
| Socio-emotional area: Negative  reactions | 10. Shows Disagreement: Any act that shows rejection of what another person has said | I don’t think this is right. |
|  | 11. Shows Tension: Any act that indicates that a person is experiencing anxiety | Time is really tight. |
|  | 12. Shows Antagonism/Seems Unfriendly: Any act that shows negative feelings toward another person | Your behavior has made me not in the mood to continue this task. |

*Note. This table reproduces the 12 categories of Bales’ Interaction Process Analysis (IPA) as summarized by Nam et al. (2009, p. 773).* *Examples are drawn from our dataset to illustrate each category in context.*
